# Spatiotemporal Insights Into RNA–Organelle Interactions in Neurons

**DOI:** 10.3389/fcell.2021.663367

**Published:** 2021-06-10

**Authors:** Shivani C. Kharod, Dong-Woo Hwang, Sulagna Das, Young J. Yoon

**Affiliations:** ^1^Department of Anatomy and Structural Biology, Albert Einstein College of Medicine, Bronx, NY, United States; ^2^Dominick P. Purpura Department of Neuroscience, Albert Einstein College of Medicine, Bronx, NY, United States

**Keywords:** mRNA transport, mRNA translation, vesicular trafficking, organellar trafficking, single molecule imaging, high-resolution imaging, neurons

## Abstract

Neurons exhibit spatial compartmentalization of gene expression where localization of messenger RNAs (mRNAs) to distal processes allows for site-specific distribution of proteins through local translation. Recently, there have been reports of coordination between mRNA transport with vesicular and organellar trafficking. In this review, we will highlight the latest literature on axonal and dendritic local protein synthesis with links to mRNA–organelle cotransport followed by emerging technologies necessary to study these phenomena. Recent high-resolution imaging studies have led to insights into the dynamics of RNA–organelle interactions, and we can now peer into these intricate interactions within subcellular compartments of neurons.

## Introduction

**FIGURE 1:**
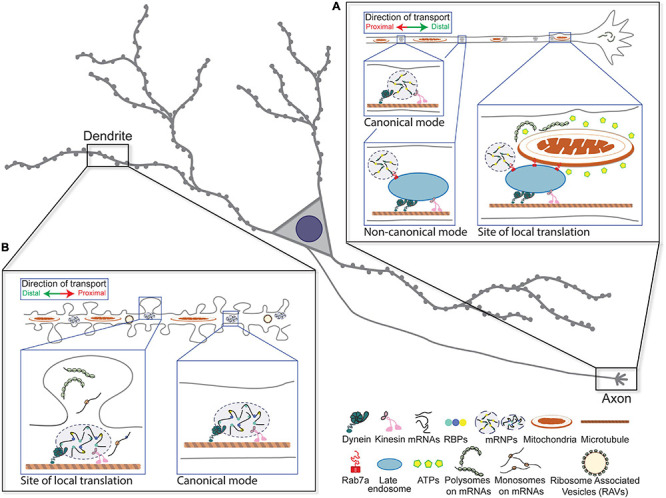
Canonical and non-canonical modes of the long-range transport of messenger ribonucleoprotein (mRNPs) in neurons. **(A)** Molecular motor-based canonical and non-canonical modes of mRNP transport in axon are depicted. Local translation of mRNAs through the interaction among mRNP, late endosome, and mitochondria at the distal site of axon is illustrated. **(B)** Molecular motor-based long-range transport of mRNPs in dendrites is indicated. Local translation in and near the base of activated spines is depicted.

Messenger RNAs (mRNAs) are distributed throughout subcellular compartments and subject to locally organized translation for the purpose of protein enrichment or sequestration. One clear advantage of transporting mRNAs is that the transcript can serve as a blueprint to rapidly produce multiple copies of the protein when and where the cell needs them. Targeting mRNAs to specific subcellular sites requires three major components. First, the *cis*-acting element(s) within the mRNA, referred to as the “localization element” or “zipcode,” are most frequently found in the 3′ untranslated region (UTR). Second, RNA-binding proteins (RBPs) function as *trans*-acting factors that recognize and bind to the *cis*-acting elements in a sequence-specific manner. Third, the resulting messenger ribonucleoprotein (mRNP) complex interacts with adaptor proteins that mediate active transport, anchoring, or translational silencing (Figure 1). Recent studies have uncovered the complexity among these components in directing localization in neurons. For example, the 3′UTR of localized mRNAs can be heterogeneous as a result of posttranscriptional processes where specific 3′UTR isoforms may localize to a subcellular compartment in a length- and sequence-dependent manner (Tushev et al., 2018). Moreover, multiple RBPs can bind to a 3′UTR, sequentially or simultaneously, and exert a combinatorial effect on localization, translation, or degradation of mRNAs (reviewed in Mayr, 2017). Therefore, the diversity of 3′UTRs and the repertoire of RBPs on a transcript may be particularly important for neuronal development and function.

Local protein synthesis in axons and dendrites has been shown to influence various forms of activity-driven changes in the synapses, commonly known as synaptic plasticity (Kang and Schuman, 1996; Martin et al., 1997; Huber et al., 2000; Miller et al., 2002; Younts et al., 2016; Monday et al., 2020 and reviewed in Nakahata and Yasuda, 2018). This modification in the physiological properties of synapses serves as a basis for experience-dependent changes in the brain, including processes like long-term memory (reviewed in Mayford et al., 2012; Monday and Castillo, 2017). While local translation has been proposed as a ubiquitous regulatory mechanism for rapid remodeling of synapses in response to external cues, not all neuronal mRNAs are evenly distributed along the processes, and not all synaptic proteins are simultaneously present at synapses. Thus, individual mRNAs may localize and translate with varying kinetics (Butko et al., 2012; Yoon et al., 2016; reviewed in Nakahata and Yasuda, 2018; Holt et al., 2019). Recent studies have found a new mode of mRNA transport, localization, and translation in axons, where organelles such as endosomes and lysosomes are involved in these events. In this mini review, we will discuss current views on mRNA–organelle interactions in neurons with focus on the single-molecule imaging approaches that enable access to spatial and temporal information at the cellular level.

## The Local Translatome at Synapses

Parsimony is a prominent feature to describe the efficiency of biological systems. For example, local translation of mRNAs is more efficient than transporting individual proteins made elsewhere to a distant location. The first line of evidence for local protein synthesis in postsynaptic compartments came from the discovery of polyribosomes in or near dendritic spines (i.e., bases of spines) of hippocampal neurons by electron microscopy (Steward and Levy, 1982; Steward, 1983). Enrichment of polyribosomes within dendritic spines of hippocampal neurons after high-frequency electrical stimulation (i.e., tetanic stimulation) provided further evidence for activity-induced local translation in postsynaptic compartments (Ostroff et al., 2002; reviewed in Harris, 2020). Later on, findings of polyribosomes and their colocalization with β-actin mRNA in the growth cones of developing hippocampal neurons suggested that translation also occurred in presynaptic compartments of neurons (Deitch and Banker, 1993; Bassell et al., 1998; Zhang et al., 1999; Hafner et al., 2019; Ostroff et al., 2019).

The identification and quantification of newly synthesized proteins have also been accomplished by the multi-omics approaches, characterized by the use of high-throughput methodologies such as mass spectrometry and mRNA sequencing. For example, newly synthesized protein can be identified when labeled with non-canonical amino acids or aminoacyl-tRNA analogs, as in the stable isotope labeling by amino acids in cell culture (SILAC) (Dörrbaum et al., 2020), bio-orthogonal non-canonical amino acid tagging (BONCAT) (Dieterich et al., 2006), or puromycin-associated nascent chain proteomics (Aviner et al., 2013). In conjunction with non-canonical amino acid tagging, mass spectrometry provides a compendium of the nascent proteome, although with limitations in compartment-type specificity and temporal resolution (Evans et al., 2020). Recent development of mouse models such as the Ribotag (Sanz et al., 2009) and the translating ribosome affinity purification (TRAP) strategies (Doyle et al., 2008; Dougherty et al., 2010) offer new ways to identify translating transcripts and complement proteomic approaches. These approaches employ cell-specific expression of tagged ribosomal proteins, which allow isolation and subsequent identification of mRNAs that are engaged with ribosomes—that is, the translatome. By analyzing physically and biochemically isolated brain regions (e.g., neuropil), the focused multi-omics approaches have finally shed light on the *local translatome* (Shigeoka et al., 2016; Ouwenga et al., 2018; Ostroff et al., 2019; Biever et al., 2020).

Although the rationale for local protein synthesis is quite compelling in neurons, many questions remain. For instance, the availability of the ribosomes and translation machinery has been highly debated (reviewed in Kosik, 2016; Biever et al., 2019). One proposed mechanism is that localized mRNAs are preferentially are translated by monosomes as opposed to polysomes, as identified by enrichment of monosome-bound mRNA populations in purified synaptic neuropil (Biever et al., 2020). However, it is not clear how the local translation of monosome-preferring transcripts is shaped and regulated, as monosome-mediated translation displays less frequent translation initiation and slower translation elongation rate, thus, suggesting an additional layer of regulation at synapses. Another question is whether membrane-bound or secreted proteins are locally made in dendrites or axons. In the canonical secretory route, membrane proteins are synthesized and exported from the endoplasmic reticulum (ER) to the Golgi (reviewed in Ramírez and Couve, 2011). For this reason, it is not clear how locally synthesized proteins can be packaged into releasable vesicles when there is no observable rough ER or well-defined Golgi apparatus in dendrites and axons. However, it has been reported that neurotransmitter receptors such as GABA_A_ receptors and NMDA-type or AMPA-type glutamate receptors can bypass the Golgi apparatus (Hanus et al., 2016). The precise contributions of each mechanism and their relevance under a specific cellular state remains unknown. These are a few among many outstanding questions on how the local translatome is maintained and regulated—that is, how an individual mRNA as part of a large dynamically regulated synapse-specific transcriptome is transported and translated at distal synapses.

## Canonical Mode of mRNA Transport

Directed transport, diffusion capture, and selective stabilization are among the strategies diverse cell types utilize to achieve mRNA localization (reviewed in Buxbaum et al., 2015). Both long-range transport of mRNPs and organelles are mediated by microtubule-based molecular motors, kinesin and dynein, in pre- and postsynaptic compartments of neurons (Prekeris et al., 1999; Park et al., 2006 and reviewed in Maday et al., 2014; Das et al., 2019; Guedes-Dias and Holzbaur, 2019) where mRNAs have been observed traveling bidirectionally at 0.5–2.0 μm/s in both axons and dendrites (Park et al., 2014; Yoon et al., 2016; Das et al., 2018; Turner-Bridger et al., 2018; Donlin-Asp et al., 2020). The polarized structure of the neuron provides an ideal system to study and characterize mRNA transport and local protein synthesis.

## Vesicle- and Organelle-Mediated mRNA Transport

Studies have indicated an alternative or non-canonical mode of mRNA transport where mRNP granules were seen cotrafficking with vesicles in axons. In contrast to earlier studies, where vesicle-coupled mRNA localization was linked to short-range movement of transcripts (reviewed in Haag et al., 2015), it now appears that mRNAs could travel long distances by hitching a ride with endosomes and lysosomes (Cioni et al., 2019; Liao et al., 2019). Notably, by docking onto moving vesicles, mRNPs may bypass the need for direct interactions with molecular motors (reviewed in Maday et al., 2014; Salogiannis and Reck-Peterson, 2017; Guedes-Dias and Holzbaur, 2019). In the fungus, *Ustilago maydis*, it has been documented that mRNAs localize to the growing tip of the hyphae by hitchhiking on endosomes (Baumann et al., 2016). It is intriguing that axons have convergently evolved to utilize vesicle-mediated cotransport of mRNA as an additional pathway to localize transcripts within axons. These observations lead to exciting mechanistic questions on whether other motile organelles traffic with mRNAs to synapses or whether this cotransport is subject to regulation by synaptic activity. In fact, high-resolution reconstructions of neurons revealed an extensive juxtaposition of membranous organelles, such as the smooth ER and the mitochondria, as well as synaptic vesicles and endosomes, implicating these structures in mRNA transport to distal synapses (Harris, 2020). Cycling endosomes and endosome-related lysosomes, while classically known for sorting, trafficking, and recycling of membrane proteins through endocytosis and protein degradation (Kennedy and Ehlers, 2006), can also participate in the long-range transport of mRNPs.

Evidence that mRNA granules are hitchhiking on motile organelles arises from a study that used fluorescence imaging and proximity labeling proteomics to reveal an association between RNA granules and lysosomes (Liao et al., 2019). By simultaneously tracking the movement of a granule marker, G3BP1, along with LAMP1-positive late endosomes or lysosomes, the authors demonstrated that RNA granules and lysosomes cotraffic in cortical neurons. Additionally, the authors uncovered a molecular tether, annexin A11, that bridges RNA granules and lysosomes. Interestingly, amyotrophic lateral sclerosis (ALS)-linked mutation in annexin A11 reduced its association with LAMP1-postive compartments in neurons, rendering the formation of more stable and possibly aggregation-prone RNA granules. In an unrelated study, an association between mRNAs and endosomes was investigated in the axons of *Xenopus* retinal ganglion cells (Cioni et al., 2019). The authors showed that local translation occurred in close proximity to Rab7a-associated late endosomes and mitochondria, suggesting that membranous organelles may serve as sites for local translation for a significant fraction of axonal mRNAs (Figure 1A; reviewed in Béthune et al., 2019; Rossoll and Bassell, 2019). About 20–30% of mRNAs that colocalized with endosomes were observed moving bidirectionally along with ribosomal proteins and RBPs, suggesting that these organelles can act as hubs to recruit components of local translation. Using the puromycylation assay, they found newly synthesized proteins associated with Rab7a-positive endosomes, which decreased when Rab7a was mutated or endosomal maturation was pharmacologically impaired (Cioni et al., 2019). Therefore, the authors concluded that a subpopulation of axonal endosomes acts as platforms of protein synthesis. Furthermore, Rab7a-positive endosomes halted or paused when they encountered mitochondria forming contacts that were maintained on the range of minutes, coincident with epochs of translation. This association mediates the synthesis of lamin B2 (Lmnb2) and voltage-dependent anion-selective channel proteins 2 (Vdac2), which have known roles in the maintenance of axonal mitochondrial integrity and function. Importantly, both reports identified a link to neurological diseases, which underscores the physiological significance of vesicle-mediated mRNA transport as means to localize transcripts in axons.

Advances in the field have brought additional outstanding questions to light. For example, the observed RNA–organelle interactions are consistent with the role of mitochondria supplying energy to meet local demands for new protein synthesis during synaptic plasticity (Rangaraju et al., 2019 and reviewed in Rossoll and Bassell, 2019). Using stimulated emission depletion (STED) microscopy to resolve mitochondrial compartments in live neurons, Rangaraju et al. (2019) found that mitochondria exist in temporally stable compartments of single or multiple mitochondrial filaments in dendrites. Of note, mRNAs found to be transported by the endosomes are transcripts for nuclear-encoded mitochondrial proteins, and yet, the extent to which nuclear-encoded mitochondria mRNAs are translated by cytosolic ribosomes in close proximity to mitochondria has not been completely surveyed. Moreover, potential advantages of endosome-mediated transport and translation over molecular motor-based mechanisms in neurons are still largely unknown (Hillefors et al., 2007; Aschrafi et al., 2012; Yoon et al., 2012; Yousefi et al., 2020, and reviewed in Rangaraju et al., 2017). Therefore, how endosomes may mediate long-range transport of specific subsets of mitochondrial mRNAs is an important question that remains to be answered. To this end, proximity-labeling methods like APEX-seq, where RNA molecules within close proximity of organelles can be assessed at the transcriptomic level, holds the promise to survey endosome-associated as well as mitochondria-associated mRNAs (Fazal et al., 2019). In conjunction with organelle-specific ribosome profiling method, it may be feasible to identify locally translated mRNAs at the contact sites between endosomes and mitochondria (Jan et al., 2014).

While vesicular and organellar mRNA transport has been better characterized in axons, a recent study characterized a newly described ER subcompartment called the ribosome-associated vesicle (RAV) in dendrites. Using multiple high-resolution microscopy techniques, the authors visualized ER network dynamics and RAVs in real time (Carter et al., 2020). Moreover, RAVs are often observed localized in close apposition to mitochondria. As the name suggests, RAVs have ribosomes attached to the cytosolic side of the vesicle that are presumably engaged in translation. Together, these studies support an emerging model where motile organelles are not solely dedicated to their physiological function but also serve as platforms or hubs to facilitate local protein synthesis. Furthermore, the goal of vesicle-mediated mRNA transport and translation is likely to replenish proteins onto the organelle, which they are tethered to. Further studies regarding the spatial and temporal nature of these interactions will provide mechanistic insight into translational control in dendrites and axons. To resolve these molecular interactions and to understand the mechanisms of local translation on organelles near synapses, high-resolution microscopy techniques paired with single-molecule imaging of mRNAs in neurons will be indispensable.

## Single-Molecule Imaging of mRNA Localization and Local Translation

With the development of orthogonal fluorescence tagging systems and new generations of bright and photostable fluorophores, modern-day single-molecule imaging can probe dynamic behaviors of an individual molecule in unprecedented detail (Grimm et al., 2015; Liu et al., 2015, and reviewed in Lavis, 2017). Direct visualization of a molecule allows for quantitative assessment of molecular behaviors in subcellular compartments of intact neurons and brain tissues (reviewed in Triller and Choquet, 2008). In particular, single-molecule imaging of mRNAs in live neurons can be designed to ask the question on how a cell parcels mRNAs out to specific synapses during synaptic plasticity by providing spatiotemporal information with nanoscale precision and subsecond resolution.

To achieve fluorescent labeling of target mRNAs in eukaryotic systems, a genetic tagging approach with the bacteriophage MS2 and PP7 RNA stem-loops and capsid proteins (MCP and PCP, respectively) has been developed (Bertrand et al., 1998; Das et al., 2018; Tutucci et al., 2018, and reviewed in Sato et al., 2020). This approach employs a two-component strategy, in which target mRNAs are tagged with MS2 or PP7 stem-loops within the 3′UTR, providing the binding sites for fluorescently labeled capsid proteins. The highly specific interaction between the coat protein and the cognate stem-loop facilitates the direct visualization of individual mRNA molecules (Lionnet et al., 2011; Park et al., 2014; Das et al., 2018; Garcia and Gregor, 2018; Tutucci et al., 2018; Lee et al., 2019). Alternatively, a hybridization-based approach with a fluorogenic oligonucleotide probe (i.e., the molecular beacon), designed to escape from self-quenching upon binding to target mRNAs, can be used for live tracking of endogenous mRNAs in neurons (Tyagi and Kramer, 1996; Turner-Bridger et al., 2018; Cioni et al., 2019; Donlin-Asp et al., 2020). A detailed and comparative review of various mRNA-tagging technologies is presented in these reviews (Braselmann et al., 2020; Sato et al., 2020; Wu and Jaffrey, 2020).

Single particle tracking and analyses of endogenous β-actin, Arc mRNAs, and exogenous reporters like Rgs4 mRNAs in neurons have revealed that long-range transport in dendrites and axons is a non-processive and intrinsically heterogeneous process, where an mRNA stochastically switches between stationary and bidirectionally moving phases (Supplementary Video 1; Park et al., 2014; Yoon et al., 2016; Das et al., 2018; Turner-Bridger et al., 2018; Bauer et al., 2019; Donlin-Asp et al., 2020). In particular, bidirectional phases are exemplified by microtubule-based outward-bound (anterograde) and inward-bound (retrograde) movements as in the case of active transport of synaptic and organelle cargos (reviewed in Maday et al., 2014; Guedes-Dias and Holzbaur, 2019), which are the product of simultaneous binding of anterograde-driving motor kinesin and retrograde-driving motor dynein. The net contribution of these motors determines the final directionality of the mRNA transport either toward distal dendrites and axon tips or soma, respectively. In particular, this commonality in the movements of mRNA and organelle transport has served as important basis for studies to identify mRNA–organelle interactions (Cioni et al., 2019; Liao et al., 2019).

In addition, a characteristic halt in mRNA movement at activated synapses is a prevailing pattern observed in neurons, which may lead to the engagement of mRNAs with local regulatory factors (Video 1). It has been postulated that anchoring of patrolling mRNAs at the base of activated synapses establishes and maintains synaptic plasticity by presumably increasing the probability for mRNA to come into contact with locally available regulators, such as the ribosome (Figure 1B; reviewed in Kiebler and Bassell, 2006; Doyle and Kiebler, 2011). In fact, when the dendritic spines are locally stimulated by uncaging glutamate, β-actin mRNAs preferentially localized at the bases of activated spines (Yoon et al., 2016). This localization occurred as early as 15 min within a segment of 6 μm with more than 50% probability. Importantly, this induced localization at the site of stimulation is correlated with enhanced local protein synthesis and subsequent actin polymerization in the corresponding spines, suggesting that local translation underlies the actin-mediated remodeling of the stimulated synapses.

Recently developed methods for visualization of translating mRNAs in living cells will provide a means to capture the moment of translation (Morisaki et al., 2016; Wang et al., 2016; Wu et al., 2016; Yan et al., 2016). Briefly, the translation reporter entails an epitope array (e.g., SunTag, MoonTag, or Spaghetti Monster) fused in-frame to the coding sequence of the reporter followed by the MS2/PP7 stem-loops, which allows simultaneous detection of nascent peptides and mRNA by fluorescently labeled antibody fragments and MS2/PP7 capsid proteins, respectively (Tanenbaum et al., 2014; Viswanathan et al., 2015; Boersma et al., 2019). In fact, cotracking of translating mRNAs along with its own nascent peptides in various cell types has uncovered detailed kinetic properties of translation; for example, a ribosome translates a given mRNA at three to five codons per second, and this computes to one to four translation initiation events per minute (Wu et al., 2016; Yan et al., 2016). Moreover, utility of this nascent peptide imaging approach has been demonstrated not only in studying various modes of translation (e.g., frameshift, nonsense-mediated decay, and miRNA-mediated translation repression) but also in the study of organelle-mediated transport and translation of mitochondria-associated mRNAs in axons (Boersma et al., 2019; Cioni et al., 2019; Hoek et al., 2019; Lyon et al., 2019; Ruijtenberg et al., 2020).

Of note, endogenous mRNA tagging methodologies with the CRISPR-mediated knock-in strategy may hold great promise as it can provide more accurate picture of the target mRNA’s molecular behavior as opposed to the overexpression of exogenous reporter mRNAs (Mikuni et al., 2016; Donlin-Asp et al., 2020; Willems et al., 2020). A limitation of exogenous reporters is that, often, the expression levels are not regulated owing to the absence of the native regulatory elements (promoters, coding regions, and UTRs) of the gene and may not represent the endogenous counterpart. Therefore, the next frontier in single-molecule imaging of mRNAs is to visualize local translation of endogenous synaptic mRNAs at activated synapses and to capture the dynamic translation events during synaptic plasticity (reviewed in Holt et al., 2019). To summarize, single-molecule imaging of mRNAs in real time has provided a wealth of spatiotemporal information about how mRNAs are transported and localized at synaptic sites, thereby, making it feasible to infer underlying regulatory mechanisms of local translation during synaptic plasticity.

## Future Perspectives on the Study of the Organelle-Mediated Local Translation

It is unclear how the interactions between mRNAs and organelles shape local translation at individual synapses. The transcriptomic approach excels at identifying mRNAs, localized and translated at synaptic sites, although detailed spatial and temporal information of mRNAs and their interactions with organelles with regard to individual synapses is often unattainable. Single-molecule imaging, on the other hand, can provide nanoscale spatial and subsecond temporal resolution of mRNAs and organelles, thus, well-suited for identifying intracellular interactions between individual mRNAs and organelles, but is inherently limited in its throughput. Nevertheless, it has yet to be shown via imaging-based approaches that the presence of an mRNA at a synapse directly implies its local translation, and current approaches do not accurately inform about the amount of protein produced from a single transcript. Therefore, methods that may bridge the gap between these two approaches will help to advance the field. This entails a large-scale direct visualization method that allows for probing the dynamics of mRNA–organelle interactions and local translation of mRNAs.

Over the last decade, as significant technology progresses in a large-scale, direct mRNA visualization methods have been made. Newly devised multiplexed single-molecule fluorescence *in situ* hybridization (smFISH) methods have shown great potential to detect hundreds to thousands of individual mRNAs at a time (Chen et al., 2015; Shah et al., 2016; Moffitt et al., 2018; Eng et al., 2019; Alon et al., 2021). For example, multiplexed error-robust fluorescence *in situ* hybridization (MERFISH) and sequential FISH (seqFISH) employ a sequential hybridization strategy, where gene-specific primary and multiple secondary fluorescent probes are subject to successive rounds of hybridizations to achieve unique combinatorial labeling of individual transcripts. Furthermore, smFISH in physically expanded tissues with polymers, dubbed expansion FISH (ExFISH), has emerged as a promising method that can achieve nanoscale spatial resolution (Chen et al., 2016; Chang et al., 2017). Importantly, the versatility of these methods to image both mRNA and protein molecules in preserved tissues provide spatial significance and *in vivo* relevance (see perspectives article, Wassie et al., 2019). When combined with protein retention expansion microscopy (proExM) and nascent peptide-imaging methods, such as SunTag, MoonTag, and/or Spaghetti Monster, these approaches may resolve the precise locations of mRNAs and corresponding nascent proteins with respect to other organelles in dendrites and axons (Tillberg et al., 2016; Gao et al., 2019). With the advent of ever-evolving high-resolution microscopy techniques and orthogonal tagging methodologies with improved fluorophore chemistry, single-molecule mRNA imaging can complement multi-omics approaches to uncover the underlying mechanisms of local translation at steady state and during synaptic plasticity.

## Author Contributions

SK and D-WH contributed equally to writing of this manuscript. SD and YY supervised and made the final edits. All authors contributed to the article and approved the submitted version.

## Conflict of Interest

The authors declare that the research was conducted in the absence of any commercial or financial relationships that could be construed as a potential conflict of interest.

## Supplementary Material

Supplementary Video 1 Real-time visualization of β-actin mRNA transport in neurons. Real-time movements of MS2 stem loop-tagged β-actin mRNA in dendrites is displayed. Discrete puncta (e.g., black dots) indicate mRNA particles. Scale bar indicates 10 μm.

## Abbreviations

mRNA: messenger RNA
UTR: untranslated region
RBP: RNA-binding protein
mRNP: messenger ribonucleoprotein
STED: stimulated emission depletion microscopy
RAVs: ribosome-associated vesicles
EMCCD: electron multiplying charge-coupled device
TRAP: translating ribosome affinity purification
smFISH: single-molecule fluorescence *in situ* hybridization
MERFISH: multiplexed error-robust fluorescence *in situ* hybridization
seqFISH: sequential FISH
ExFISH: expansion FISH
proExM: protein retention expansion microscopy.
